# Genome-wide analysis of the *ERF* Family in *Stephania japonica* provides insights into the regulatory role in Cepharanthine biosynthesis

**DOI:** 10.3389/fpls.2024.1433015

**Published:** 2024-09-04

**Authors:** Hanting Yang, Baimei Liu, Haiyan Ding, Zhaoyu Liu, Xiaodong Li, Tianxing He, Ya Wu, Yuxuan Zhang, Can Wang, Liang Leng, Shilin Chen, Chi Song

**Affiliations:** ^1^ School of Pharmacy/School of Modern Chinese Medicine Industry, Chengdu University of Traditional Chinese Medicine, Chengdu, China; ^2^ Institute of Herbgenomics, Chengdu University of Traditional Chinese Medicine, Chengdu, China; ^3^ School of Chinese Materia Medica, Tianjin University of Traditional Chinese Medicine, Tianjin, China; ^4^ Wuhan Botanical Garden, Chinese Academy of Sciences, Wuhan, China

**Keywords:** *ERF*, *Stephania japonica*, Cepharanthine biosynthesis, expression patterns, genome-wide analysis

## Abstract

**Introduction:**

Cepharanthine (CEP), a bisbenzylisoquinoline alkaloid (bisBIA) extracted from *Stephania japonica*, has received significant attention for its anti-coronavirus properties. While ethylene response factors (ERFs) have been reported to regulate the biosynthesis of various alkaloids, their role in regulating CEP biosynthesis remains unexplored.

**Methods:**

Genome-wide analysis of the ERF genes was performed with bioinformatics technology, and the expression patterns of different tissues, were analyzed by transcriptome sequencing analysis and real-time quantitative PCR verification. The nuclear-localized ERF gene cluster was shown to directly bind to the promoters of several CEP-associated genes, as demonstrated by yeast one-hybrid assays and subcellular localization assays.

**Results:**

In this work, 59 SjERF genes were identified in the *S. japonica* genome and further categorized into ten subfamilies. Notably, a SjERF gene cluster containing three *SjERF* genes was found on chromosome 2. Yeast one-hybrid assays confirmed that the SjERF gene cluster can directly bind to the promoters of several CEP-associated genes, suggesting their crucial role in CEP metabolism. The SjERFs cluster-YFP fusion proteins were observed exclusively in the nuclei of *Nicotiana benthamiana* leaves. Tissue expression profiling revealed that 13 *SjERFs* exhibit high expression levels in the root, and the qRT-PCR results of six *SjERFs* were consistent with the RNA-Seq data. Furthermore, a co-expression network analysis demonstrated that 24 *SjERFs* were highly positively correlated with the contents of various alkaloids and expression levels of CEP biosynthetic genes.

**Conclusion:**

This study provides the first systematic identification and analysis of ERF transcription factors in the *S.japonica* genome, laying the foundation for the future functional research of SjERFs transcription factors.

## 1 Introduction

The COVID-19 outbreak in 2019 had a severe global impact, prompting scientists worldwide to collaborate in the search for effective drugs (Li et al., 2022; Yang et al., 2024a). Cepharanthine (CEP) has demonstrated the ability to inhibit the entry of SARS-CoV-2 into cells by blocking the virus’s attachment to its intended target cells (Kumar et al., 2022). This characteristic makes CEP a promising therapeutic agent for potential anti-COVID-19 treatments (Kumar et al., 2022; Fan et al., 2020). CEP, a bisBIA isolated from *Stephania japonica*, with the biological activities of antioxidant (Chen et al., 2019), antitumor (Zhang et al., 2021), and immunomodulatory (Xu et al., 2021). CEP predominantly accumulates in the roots of *S. japonica*, followed by the leaves and stems (Leng et al., 2024). *S. japonica* (Thunb.) Miers, a tangled deciduous woody vine belonging to the Menispermaceae family and *Stephania* genus (Al-Amin et al., 2022), is commonly used in traditional Chinese folk medicine for its heat-clearing, detoxifying, and “wind and blockage” dispelling properties in the human body (Xiao et al., 2019). Given the increasing clinical demand for CEP, it is crucial to investigate its biosynthesis and transcriptional regulation.

The biosynthesis of CEP primarily initiates with dopamine and 4-hydroxyphenyl acetaldehyde catalyzed by norclaurane synthesis (NCS) (Minami et al., 2007), norclaurane 6-O-methyltransferase (6OMT) (Li et al., 2020), coclaurine N-methyltransferase (CNMT) (Zhao et al., 2020), and undergoes multi-step reaction catalyzed by *OMT/CYP80A* (Stadler et al., 1988; Carina and Till, 2019). The oxidase *CYP80A1* selectively couples two N-methylcocoyl units in their benzylic portion, forming the simplest bisBIA (Kraus and Kutchan, 1995). The biosynthesis of guattegaumerine and berbamunine of bisBIA s has been elucidated (Payne et al., 2021). In *S. japonica*, *SjNCS2* and *SjNCS4* possessed *NCS* functionality and exhibited superior enzymatic activities compared with the *Coptis chinensis NCS* (Leng et al., 2024). However, the downstream biosynthetic pathways of CEP remain unclear (**Supplementary Figure S1**).

*ERF* transcription factors are significant regulators in various plant biological processes, including alkaloid biosynthesis (Feng et al., 2020; Yamada and Sato, 2021). For instance, clustered ORCA transcription factors (ORCA2-6) regulate the expression of different monoterpene indole alkaloid biosynthetic genes in *Catharanthus roseus* (Paul et al., 2020; Singh et al., 2020). In *Nicotiana benthamiana*, *NtERF189* acts as a master regulator of nicotine biosynthesis by recognizing GCC-box-like elements in the promoter of nicotine biosynthetic genes (Shoji et al., 2010; Shoji and Hashimoto, 2012). *OpERF*2 positively regulates the anti-cancer camptothecin biosynthesis in *Ophiorrhiza pumila* (Udomsom et al., 2016). In *Eschscholzia californica*, a luciferase reporter assay indicated that four Group IX *AP2/ERF* TFs, known as *EcERF2*, *EcERF3*, *EcERF4*, and *EcERF12*, can trans-activate *Ec6OMT* and *EcCYP719A5*, which are involved in BIA biosynthesis (Yamada et al., 2020). Transiently overexpressing *PhERF1* in petunia leaves has an impact on the production of petuniolides and petuniaserones (Shoji et al., 2023). Overexpression of ScAPD1-like significantly increased the metabolites of the phenylpropanoid pathway by directly regulating the abundance of *ScPAL* and *ScC4H* transcripts (Li et al., 2023). The ERF transcription factor WAX INDUCER1 (WIN1) promotes the accumulation of total polyphenols in *Nicotiana tabacum*, including chlorogenic acid (He et al., 2024). However, a study on the *ERF* family in *S. japonica* that regulates the biosynthesis of bisBIA has yet to be reported.

An increasing number of medicinal plant genomes have been published, including *Artemisia argyi*, *Mentha suaveolens*, and *C. roseus*, which will provide a foundation for the identification of *ERF* families and functional genomics research (Chen et al., 2023; Yang et al., 2024b; Sun et al., 2023; Pei et al., 2024). ERF protein identification and characterization have been studied in various plant species, including *Arabidopsis thaliana* (Nakano et al., 2006), barley (Taketa et al., 2008), *Fagopyum Tataricum* (Liu et al., 2019), grape (Zhuang et al., 2009; Zhu et al., 2019), apple (Girardi et al., 2013), and ginger (Xing et al., 2021). The number of *ERF* TFs family in many plants are as follows: 136 (*Oryza sativa*), 122 (*A. thaliana*), 96 (*Citrus junos*), 92 (*Camptotheca acuminata*), 80 (*Vitis vinifera*), and 60 (*E. californica*). Genome-wide identification of *ERF* transcription factor and its significance in CEP biosynthesis have not been elucidated in *Stephania* plants. This study proved the systematic identification and analysis of 59 *SjERF*s in the *S. japonica* genome using a set of bioinformatics tools. Meanwhile, tissue expression profiling and co-expression analysis of *SjERF*, CEP biosynthetic genes, and BIAs metabolites were also conducted. Yeast one-hybrid assays indicated that the *SjERFs* cluster recognizes GCC boxes in the promoters of several CEP-associated genes. This work provides valuable insight into the roles of *ERF* transcription factors in CEP biosynthesis and enhances our understanding of the *ERF* gene family in plants.

## 2 Materials and methods

### 2.1 Plant materials

The *S. japonica* plants were cultivated and harvested in Wuhan, Hubei Province, China. Different tissues of *S. japonica* including stems, leaves, roots, and shoots, were collected for transcriptome sequencing and quantitative real-time polymerase chain reaction (qRT-PCR) experiments. Three biological replicates were conducted for each experiment.

### 2.2 Identification of *SjERF* genes in the *S. japonica* genome

Our research group has acquired the genome data of *S. japonica*., and has been archived under the China National GeneBank DataBase (CNGBdb) accession number CNP0003595 (https://db.cngb.org/search/?q=CNP0003595) (Leng et al., 2024). The *AtERFs* protein sequences of *A. thaliana* were downloaded from the *Arabidopsis* Information Resource (TAIR) database (http://www.arabidopsis.org/). The hidden Markov model (HMM, PF00847) was used to search for *ERF* candidate genes in the *S. japonica* genome, with a threshold of 0.01. Furthermore, to ensure the comprehensive identification of *SjERF* genes, 121 AtERF proteins were used to BLAST the *S. japonica* protein database for ERF-containing sequences (**Supplementary Table S1**), minimizing the risk of missing any *SjERF* genes. Then, candidate proteins with only one AP2 domain were manually screened (Sakuma et al., 2002; Riechmann and Meyerowitz, 1998). The Molecular weight (MW) and pI of *SjERF* proteins were analyzed using the Expasy website (https://prosite.expasy.org/). Finally, the subcellular localization of *SjERF*s was predicted using WoLF PSORT and CELLO online software (Horton et al., 2007).

### 2.3 Classification, gene structure, and protein motif analysis of *SjERF* genes in *S. japonica*

To explore different biological characteristics and evolutionary relationships of *SjERF* proteins in *S. japonica*, an unrooted phylogenetic tree of *ERF*s protein sequences (59 *SjERFs* and 121 *AtERFs*) from *S. japonica* and *A. thaliana* was constructed by MEGA11 with 1,000 bootstrap replicates (Tamura et al., 2021). Then, an evolutionary tree was beautified and decorated using Evolview (Zhang et al., 2012). The conserved motifs of *SjERF*s protein were identified using the MEME website (parameters: number of motifs: 10, wide: 10-50, others are default values) (Bailey et al., 2009). Gene structure and protein motif of *SjERF*s were visualized using TBtools (Chen et al., 2020).

### 2.4 Analysis of *cis*-elements, microsynteny, and evolutionary patterns of *SjERF* genes

The promoter sequences of the 59 *SjERF*s (-2,000 to -1 bp) were extracted using TBtools. Subsequently, *cis*-acting regulatory elements of *SjERFs* gene promoters have been predicted and identified by PlantCARE (Lescot et al., 2002). The chromosomal positions of *SjERF* genes were retrieved from the *S. japonica* genome database and graphically represented using TBtools software. The duplication events of the *SjERF*s were analyzed using MCScanX and BLASTP (Wang et al., 2012). The synonymous relationship between *SjERFs* and *AtERFs*, *OsERFs*, *CrERFs*, and *NtERFs* was analyzed and visualized by TBtools software. The genome data of *O. sativa*, *C. roseus*, and *N. tabacum* were retrieved from the National Center for Biotechnology Information (NCBI: https://www.ncbi.nlm.nih.gov/), respectively.

### 2.5 Chromosome structure prediction and cluster prediction

The Topologically Associated Domains (TADs) were identified based on previous reports (Sun et al., 2020). Initially, the Hi-C read pairs were aligned to the *S. japonica* genome, and contact matrixes were generated using HiC-Pro (Servant et al., 2015). Subsequently, the Hi-C contact matrixes were imported into HiCExplorer (Wolff et al., 2018) and converted using the built-in function (hicConvertFormat). Then, the contact matrixes were normalized using hicNormalize with the KR correction method and corrected using hicCorrectMatrix with a filter threshold of -1.5 to 5. Next, the hicFindTADs algorithm was applied to identify TADs at various resolutions. The specific parameters used for this analysis were a minimum depth of 5, maximum depth of 10, step size of 2, and a threshold for comparisons set at 0.01.

### 2.6 Yeast one-hybrid assays

Yeast one-hybrid assays were performed to determine whether SjERF9-11 could bind to the GCC motif. The functional protein sequences of CEP biosynthetic genes with known functions were retrieved from the NCBI database, including *NCS*, *6OMT*, and *CNMT* (**Supplementary Table S2**). The candidate genes involved in CEP biosynthesis were predicted using BLASTP (option: e-value 1e^−10^). Subsequently, the functional proteins and candidate genes were used to construct phylogenetic trees with 1,000 bootstrap replicates. Additionally, the ERF binding elements in CPE biosynthetic gene promoters were predicted using PlantCARE software. The open reading frame (ORF) fragment of *SjERF9-11* was individually cloned into the effector plasmid pB42AD. Additionally, the triple tandem copy of the GCC motif (GCCGCC) or the ERF binding element from CEP-biosynthetic gene promoters was inserted into the reporter plasmid pLacZ. The effector and reporter plasmids were jointly transformed into the yeast strain EGY48 and grown on SD/-Ura/-Trp medium. Subsequently, the co-transformed cells were assayed on SD/-Ura/-Trp medium containing 5-bromo-4-chloro-3-indolyl-β-D-galactopyranoside (X-gal) for 24 hours, as previously described (Wang C. et al., 2022). Empty plasmids (pB42AD and pLacZ) were used as a negative control for the transformation. All primers utilized in this study are provided in **Supplementary Table S3**.

### 2.7 Subcellular localization

To analyze the subcellular localization of three *SjERFs*, the *SjERF9-11* ORF fragments were amplified and individually integrated into the modified plant expression vector pHB-YFP. The plasmids pHB-*SjERFs*-YFP and the empty vector pHB-YFP (serving as the negative control) were introduced into the *Agrobacterium tumefaciens* strain GV3101 and transiently infected the epidermal cells of 5-week-old *N. benthamiana* leaves, as previously described (Wang C. et al., 2021; Hao et al., 2023). YFP signals were analyzed 48 h post-infection using an LSM880 confocal laser microscope (Carl Zeiss, Germany). Nuclei were stained with, 4’6-diamidino-2-phenylindole (DAPI, Sigma, Code No. D9542). Three biological replicates were performed as reported previously, to ensure the reliability of the results.

### 2.8 RNA-seq and qRT-PCR

For RNA-seq analysis, qualified RNA samples underwent testing for database establishment. The quality of the constructed library was assessed using an Agilent 2100 Bioanalyzer, while sequencing was performed using DNBSEQ technology. All raw sequencing data have been deposited under the National Center for Biotechnology Information (NCBI) GenBank accession number PRJNA888087. The expression pattern of *SjERF*s in different tissues was analyzed by TBtools according to the FPKM values. Total RNA was extracted from the roots, stems, leaves, and shoots of *S. japonica* using a plant total RNA Extraction Kit (Foregene Biotech, Chengdu, China, Code No. RE-05011). Subsequently, reverse transcription was carried out according to the instructions provided with the gDNA Eraser reagent Kit (Foregene Biotech, Chengdu, China, Code No. RT-01032) for qRT-PCR analysis. The qRT-PCR was carried out according to previous reports, and three biological replicates were conducted for each experiment. For qRT-PCR normalization, *SjGAPDH*, a housekeeping gene in *S. japonica*, was employed as an internal control of all samples (Yang et al., 2023; Jain et al., 2018; Barber et al., 2005). The specific primers used for the analysis are detailed in **Supplementary Table S3**. The relative expression levels of *SjERFs* across various tissues were determined using the 2^−ΔΔCt^ method.

### 2.9 Co-expression network of *SjERFs* involved in CEP biosynthesis pathway

46 *SjERFs* and 9 CEP biosynthetic genes all exhibiting FPKM values exceeding 1, underwent co-expression analysis using Pearson’s correlation test. We employed untargeted metabolomics to profile BIAs across various tissues of the *S. japonica* (Leng et al., 2024). According to the expression patterns of *SjERFs*, two BIA precursors, alongside 23 BIA-type structures in roots, stems, and leaves of *S. japonica*, the partial correlation coefficient (PCC) method was used to calculate the Pearson correlation coefficient. The co-expression network of *SjERFs*, CEP biosynthetic genes, and BIAs metabolites was exhibited using Cytoscape, with the following parameters: absolute value of correlation coefficient > 0.9 and *p*-value < 0.05 (Shannon et al., 2003). The correlations between *SjERFs*, CEP biosynthetic genes, and BIAs metabolites were displayed in cluster heatmap using TBtools software.

## 3 Results

### 3.1 Genome-wide identification of 59 *SjERF* TFs in *S. japonica* genome

59 non-redundant *SjERF* genes have been identified in the *S. japonica* genome using HMMER and BLAST (**Table 1**). All identified *ERF* genes in *S. japonica* were named *SjERF1- SjERF59* according to their chromosome distribution (Huang et al., 2020). All *SjERFs* were then manually confirmed by CDD’s online software and a Simple Modular Architecture Analysis Tool (SMART) for the presence of a core domain (**Supplementary Figure S2**). The CDS sequence length of *SjERF* genes was between 605 bp (*SjERF*21) and 1305 bp (*SjERF29*), encoding 202–434 amino acids (**Supplementary Table S4**). The molecular weight (Mw) of *SjERFs* ranged from 22.58 kDa (*SjERF21*) to 47.55 kDa (*SjERF29*), with theoretical pI values ranging from 4.53 (*SjERF19*) to 10.00 (*SjERF37*). Almost all *SjERF*s were predicted to be located in the nucleus, only *SjERF*57 was located in the cytoplasmic (**Table 1**).

**Table 1:** Sequence characteristics of 59 *SjERF*s.

ID	Gene name	Type	Chr	Start	end	Strand	No. of Exon	CDS length	Mw (Da)	pI	Loc
*SjapChr1G00001760.1*	*SjERF1*	I	chr1	2711408	2714932	+	1	1194	43737.41	5.24	Nuc
*SjapChr1G00003160.1*	*SjERF2*	VIII	chr1	4557505	4557987	+	1	486	16797.81	9.51	Nuc
*SjapChr1G00003930.1*	*SjERF3*	IX	chr1	5417393	5417956	+	1	567	20593.55	6.67	Nuc
*SjapChr1G00008120.1*	*SjERF4*	V	chr1	11728956	11729894	–	1	942	34746.61	5.65	Nuc
*SjapChr1G00012460.1*	*SjERF5*	III	chr1	19185052	19189635	+	2	669	24567.25	5	Nuc
*SjapChr1G00015200.1*	*SjERF6*	V	chr1	23733363	23736700	–	2	1077	39871.19	5.22	Nuc
*SjapChr1G00029910.1*	*SjERF7*	VIII	chr1	69410464	69416430	–	1	741	27653.31	9	Nuc
*SjapChr2G00042580.1*	*SjERF8*	III	chr2	4844300	4845016	+	1	720	25621.04	5.14	Nuc
*SjapChr2G00045100.1*	*SjERF9*	IX	chr2	9308650	9309432	–	1	786	28946.91	4.66	Nuc
*SjapChr2G00045110.1*	*SjERF10*	IX	chr2	9405916	9406914	–	1	999	36667.17	8.77	Nuc
*SjapChr2G00045130.1*	*SjERF11*	IX	chr2	9534735	9535544	+	1	813	28876.08	9.40	Nuc
*SjapChr2G00053160.1*	*SjERF12*	I	chr2	41808331	41809793	–	1	1008	36626.76	8.98	Nuc
*SjapChr2G00054830.1*	*SjERF13*	III	chr2	46323345	46324282	–	1	510	18666.3	8.64	Nuc
*SjapChr2G00059370.1*	*SjERF14*		chr2	56313792	56319526	+	6	684	25387.47	9.16	Nuc
*SjapChr2G00063270.1*	*SjERF15*	IX	chr2	63234204	63235384	–	1	876	32963.44	6.13	Nuc
*SjapChr2G00063280.1*	*SjERF16*	IX	chr2	63280657	63285641	–	1	438	15963.47	5.74	Nuc
*SjapChr2G00064000.1*	*SjERF17*	VIII	chr2	64448707	64449231	+	1	528	18808.37	9.88	Nuc
*SjapChr2G00067800.1*	*SjERF18*	VIII	chr2	70373807	70374925	+	1	1122	41653.22	5.75	Nuc
*SjapChr3G00074020.1*	*SjERF19*	VII	chr3	4479441	4480956	+	2	720	26910.39	8.92	Nuc
*SjapChr3G00074030.1*	*SjERF20*	VII	chr3	4505717	4508080	+	2	774	29030.07	5.08	Nuc
*SjapChr3G00075730.1*	*SjERF21*	II	chr3	6760242	6765099	+	1	648	23872.71	5.55	Nuc
*SjapChr3G00085580.1*	*SjERF22*	IX	chr3	39428058	39428629	+	2	465	17471.53	6.11	Nuc
*SjapChr3G00085600.1*	*SjERF23*	IX	chr3	39473034	39473534	–	1	504	18653.14	5.63	Nuc
*SjapChr3G00085660.1*	*SjERF24*	VIII	chr3	39611535	39611924	–	1	393	14472.99	5.93	Nuc
*SjapChr3G00085680.1*	*SjERF25*	IX	chr3	39818424	39819128	+	1	708	26528.49	5.65	Nuc
*SjapChr3G00087630.1*	*SjERF26*	I	chr3	44029745	44030869	+	1	1128	41945.4	8.99	Nuc
*SjapChr4G00102600.1*	*SjERF27*	IV	chr4	7719916	7721567	–	2	561	20506.74	9.94	Nuc
*SjapChr4G00109960.1*	*SjERF28*	V	chr4	20749186	20749883	–	2	579	21130.05	8.98	Nuc
*SjapChr4G00119750.1*	*SjERF29*	II	chr4	54210503	54212473	–	1	627	23255.42	4.53	Nuc
*SjapChr4G00120260.1*	*SjERF30*	VII	chr4	55128464	55131323	–	2	1167	42775.32	5.08	Nuc
*SjapChr5G00123460.1*	*SjERF31*	III	chr5	3514506	3515081	+	1	579	20654.48	5.16	Nuc
*SjapChr5G00123480.1*	*SjERF32*	III	chr5	3559387	3560297	–	1	612	22578.62	6.53	Nuc
*SjapChr5G00128870.1*	*SjERF33*		chr5	12976168	12982139	–	7	1131	41961.31	6.43	Nuc
*SjapChr5G00130040.1*	*SjERF34*	V	chr5	16586160	16587790	+	2	1137	41024.09	5.96	Nuc
*SjapChr5G00131070.1*	*SjERF35*	IV	chr5	20244188	20245033	–	1	849	32297.1	6.03	Nuc
*SjapChr5G00138660.1*	*SjERF36*	VIII	chr5	45874943	45875620	+	2	597	21668.48	5.49	Nuc
*SjapChr5G00141910.1*	*SjERF37*	IV	chr5	50758866	50759834	–	1	972	35602.09	6.78	Nuc
*SjapChr5G00142510.1*	*SjERF38*	IV	chr5	51470041	51474131	–	3	1020	36785.04	9	Nuc
*SjapChr5G00143880.1*	*SjERF39*	VI	chr5	53358292	53359068	+	1	780	29876.48	7.71	Nuc
*SjapChr6G00156070.1*	*SjERF40*	V	chr6	35351515	35352727	–	2	1110	40906.17	4.82	Nuc
*SjapChr6G00166910.1*	*SjERF41*	X	chr6	52402579	52406263	+	2	867	31918.89	6.52	Nuc
*SjapChr7G00173600.1*	*SjERF42*	II	chr7	8526747	8528220	–	1	489	18343.55	6.84	Nuc
*SjapChr7G00180830.1*	*SjERF43*	III	chr7	33074754	33075338	–	1	588	21210.32	4.93	Nuc
*SjapChr7G00186210.1*	*SjERF44*	X	chr7	47780599	47783481	+	2	1308	47552.22	6.05	Nuc
*SjapChr7G00187000.1*	*SjERF45*	VIII	chr7	48993478	48993924	–	1	450	16890.64	6.37	Nuc
*SjapChr7G00187590.1*	*SjERF46*	I	chr7	49815906	49817084	+	1	1182	43619.62	6.76	Nuc
*SjapChr8G00192880.1*	*SjERF47*	V	chr8	4263704	4264879	–	2	651	23484.28	9.07	Nuc
*SjapChr8G00193970.1*	*SjERF48*	VIII	chr8	5595150	5598496	+	2	843	30641.97	9.07	Nuc
*SjapChr8G00196850.1*	*SjERF49*	II	chr8	9861774	9862899	–	1	639	23020.41	4.84	Nuc
*SjapChr8G00207160.1*	*SjERF50*	V	chr8	44219929	44220864	–	1	939	33255.74	9.6	Nuc
*SjapChr8G00207340.1*	*SjERF51*	III	chr8	44607041	44608247	–	1	675	24660.28	5.14	Nuc
*SjapChr9G00214400.1*	*SjERF52*	III	chr9	2802755	2804156	–	1	570	20702.11	5.3	Nuc
*SjapChr9G00215550.1*	*SjERF53*	II	chr9	4472534	4474762	–	2	630	23032.85	10	Nuc
*SjapChr9G00224540.1*	*SjERF54*	IX	chr9	37203805	37204545	+	1	744	26893.47	6.59	Nuc
*SjapChr9G00224550.1*	*SjERF55*	IX	chr9	37238076	37238624	–	1	552	20025.39	6.41	Nuc
*SjapChr9G00228980.1*	*SjERF56*	II	chr9	45336446	45336937	+	1	513	19254.35	8.55	Nuc
*SjapChr10G00242090.1*	*SjERF57*	VII	chr10	32748182	32748913	–	1	735	28138.51	5.09	Nuc
*SjapChr10G00244770.1*	*SjERF58*	VI-L	chr10	39579633	39581894	+	1	1014	37430.77	5.44	Cyt
*SjapChr11G00256330.1*	*SjERF59*	I	chr11	8273391	8274458	+	1	1071	40157.51	7.8	Nuc

Loc, Subcellular location; Nuc, Nucleus; Cyt, Cytoplasmic.

### 3.2 Phylogenetic relationship of *SjERF* genes

**Figure 1:**
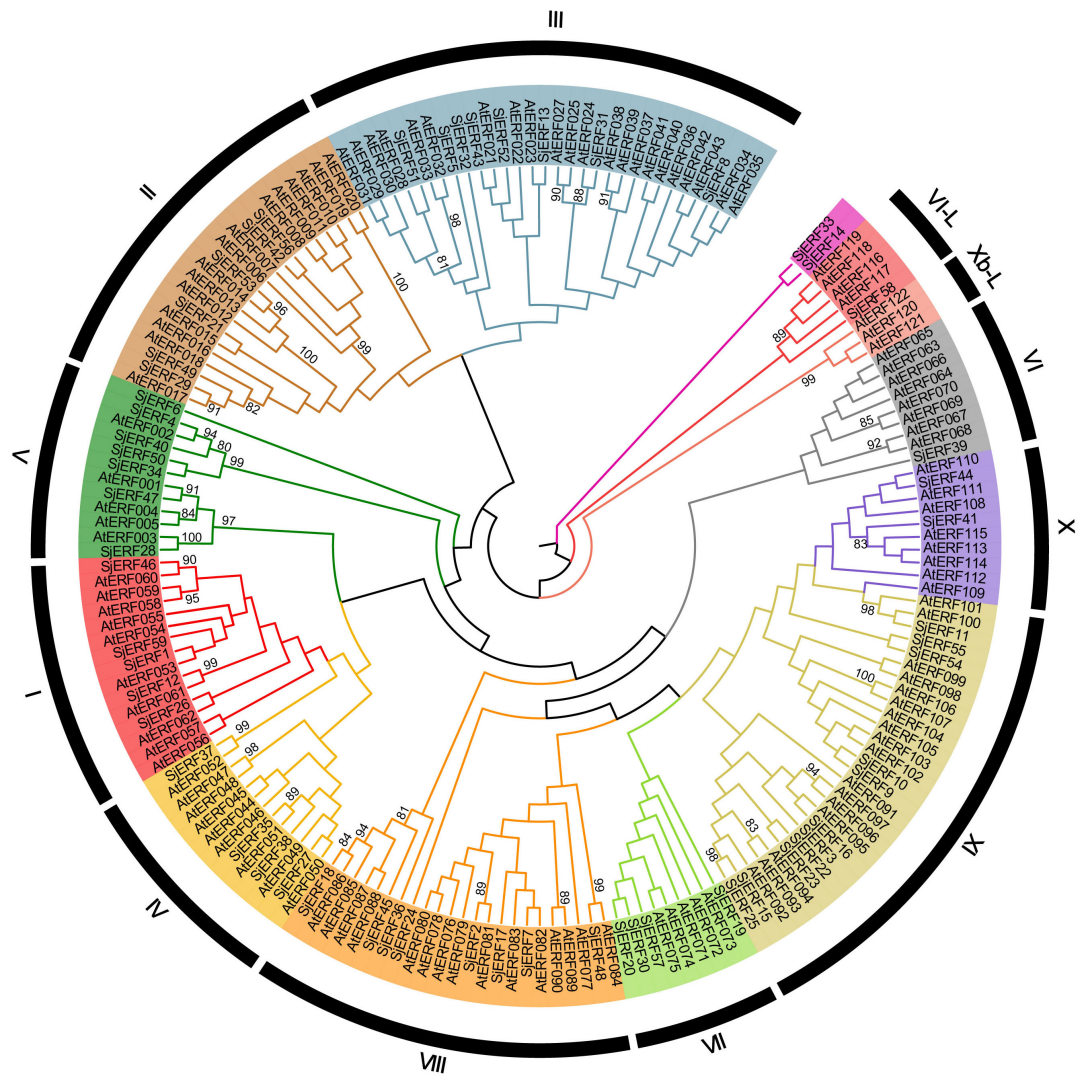
Phylogenetic tree of 59 *SjERFs* and 121 *AtERFs*. The ERF protein sequences of *S. japonica* and *A. thaliana* were used to construct the phylogenetic tree using the Neighbor-Joining (NJ) method, with 1,000 bootstrap replicates.

The unrooted phylogenetic tree of 59 *SjERFs* and 121 *AtERFs* has been constructed to explore the evolutionary relationship. 59 *SjERFs* have been divided into 10 subgroups, namely, groups I to X. A previous study has further divided the ERF family into ERF and CBF/DREB subfamily, and the ERF subfamily always classified into six groups (B1 to B6) (Zhang et al., 2015). In this analysis, group I to IV belong to the DREB subfamily, and group IV to X, and VI-L belong to the ERF subfamily, and there is no *SjERF* in group Xb-L. *SjERF1*, 12, 26, 46, and 59 were branched into group I, *SjERF21*, 29, 42, 49, 53, and 56 were branched into group II, group IX was the largest group with 11 members (*SjERF3*, 9, 10, 11, 15, 16, 22, 23, 25, 54, 55). As shown in **Figure 1**, group VI was the smallest with *SjERF39*, *SjERF58* belongs to group VI-L, and *SjERF14* and *SjERF33* don’t belong to any subfamily.

### 3.3 Gene structure and motif analysis of *SjERFs* in *S. japonica* genome

**Figure 2:**
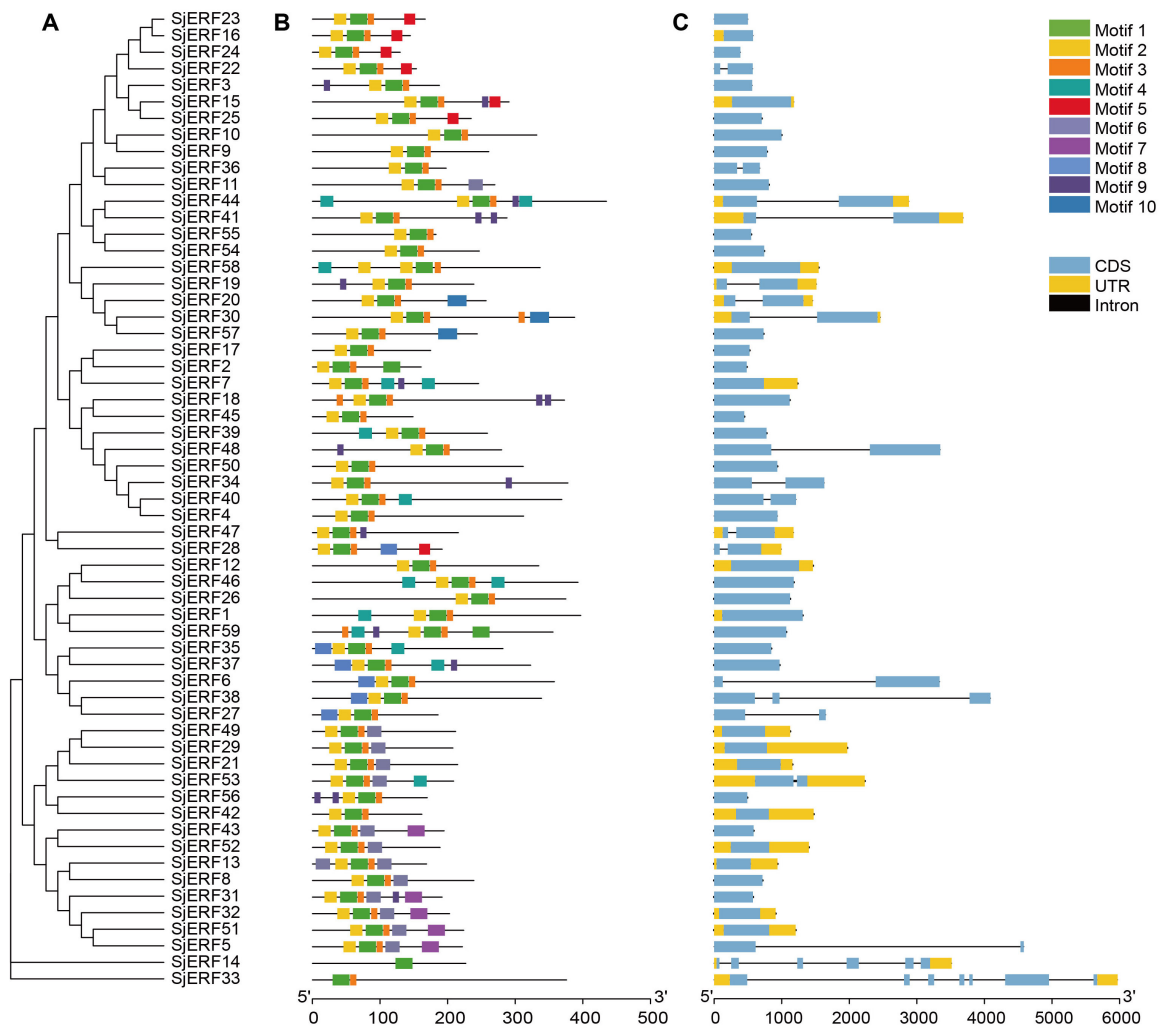
Schematic diagram of phylogenetic analysis, exon/intron distribution, and motifs analysis of *SjERF* TFs. **(A)** Phylogenetic tree of 59 SjERF proteins. **(B)** Motif distribution of 59 SjERF proteins. **(C)** The exon-intron structure of 59 *SjERF* genes. Yellow rectangle: UTR; black line: intron; blue rectangle: CDS.

To better understand the evolution and structural diversity of the *S. japonica ERF* family, the MEME (Multiple Em for Motif Elicitation) was used to analyze the conserved sequence of the 59 *SjERFs* protein. The basic information (width and best possible match sequence) of the consensus sequences of these motifs are shown in **Supplementary Table S5**. The frequent motifs of *SjERF*s are motif1 (RVWLGTFDTAEEAARAYDEAAFKLRG), motif2 (YRGVRQRPWGKWVAEIRDP), and motif3 (SKAKLNFPEE). The results showed that each motif contained 10-29 kinds of amino acids, and each *SjERF* contained motif1. Almost all *SjERFs* contain motif2, only *SjERF14* and *SjERF33* don’t belong to any subfamily that does not contain motif2, while they only contain one conserved motif (motif1), and *SjERF33* had two conserved motifs (motif1, motif3). *SjERF44*, *SjERF18*, *SjERF37*, *SjERF31*, *SjERF59*, and *SjERF7* had six conserved motifs. 59 *SjERF*s contained *ERF* conservative domain (**Figure 2B**; **Supplementary Figure S3**). Additionally, these different motif patterns show their degree of deviation among different groups. For example, motif 5 is the representative of group IX. Motif 7 is only found in group III. Motif 6 is unique to group II and VII (**Figures 2A**, **B**).

In this study, gene structures of 19 *SjERF*s (*SjERF5, 6, 14, 19, 20, 22, 27, 28, 30, 33, 34, 36, 38, 40, 41, 44, 47, 48, 53*) have one intron, and *SjERF38*, *SjERF33*, and *SjERF14* contained two or more introns. The other 40 *SjERFs* contain only one exon and no intron, accounting for 67.8% of the total *SjERF*s in *S. japonica*. Most *SjERF* genes contained only one exon in group I, II, III, and IX, except for *SjERF53*, *SjERF5*, and *SjERF22*. The unnamed subfamily genes (*SjERF14*, *SjERF33*) have 6 and 7 exons (**Figure 2C**).

### 3.4 Analysis of *cis*-acting elements in the *SjERF* genes promoter

**Figure 3:**
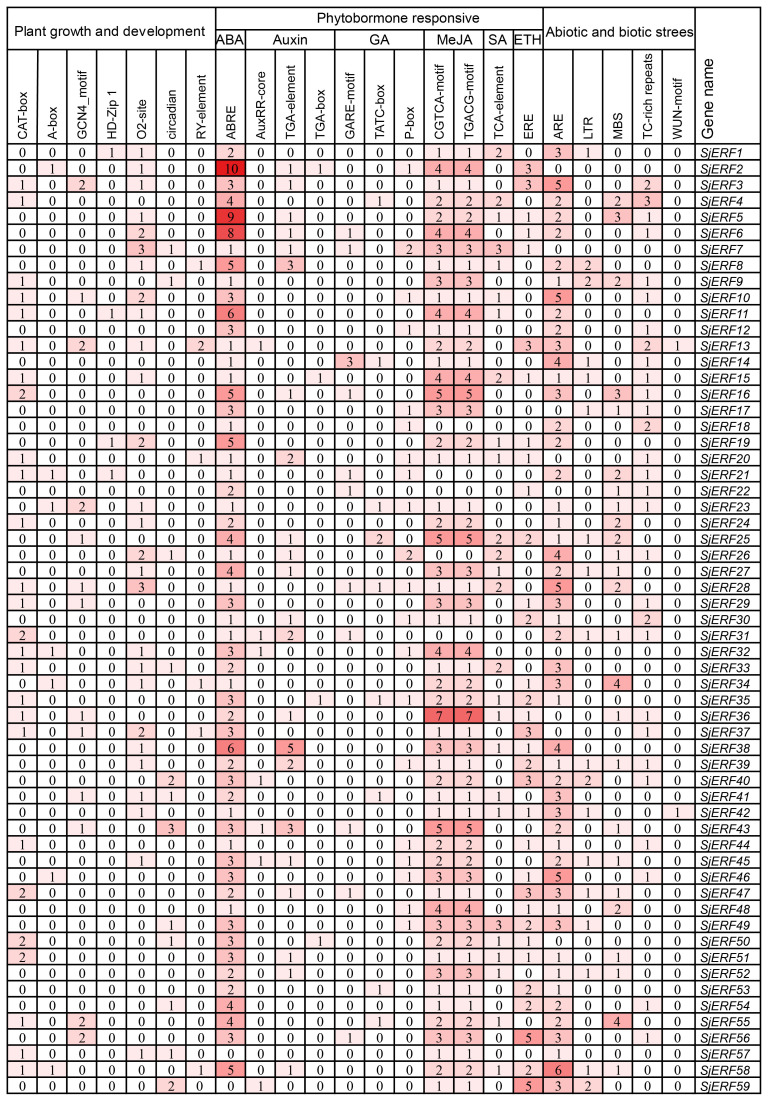
Pivotal *cis−*elements in the promoter of *SjERF* TFs.

The study identified various *cis-*acting elements located in the promoter regions of *SjERFs*, with the majority participating in hormone responses, and abiotic and biotic stress. In plant growth and development, 31 CAT-boxes were implicated in meristem expression across 26 *SjERFs* promoter regions, while 7 A-boxes participated in meristem expression in 7 *SjERFs* promoter regions (**Figure 3**; **Supplementary Table S6**). Furthermore, 18 GCN4_motif, 4 HD-Zip, 36 O_2_-site, 16 circadian control elements, and 7 seed-specific regulation elements were identified in the promoter regions of *SjERFs* (**Supplementary Figure S4**). In hormone responses, various *cis-*acting regulatory elements were identified, including 164 ABRE, 33 TGA-element, 7 AuxRR-core, 4 TGA-box, 13 GARE-motif, 10 TATC-box, 22 P-box, 123 CGTCA and TGACG-motif. However, the abiotic and biotic stress *cis-*acting elements were not found in the promoter regions of *SjERF*2, 7, 32, and 50.

### 3.5 Chromosome distribution and synteny analysis of *SjERFs*

**Figure 4:**
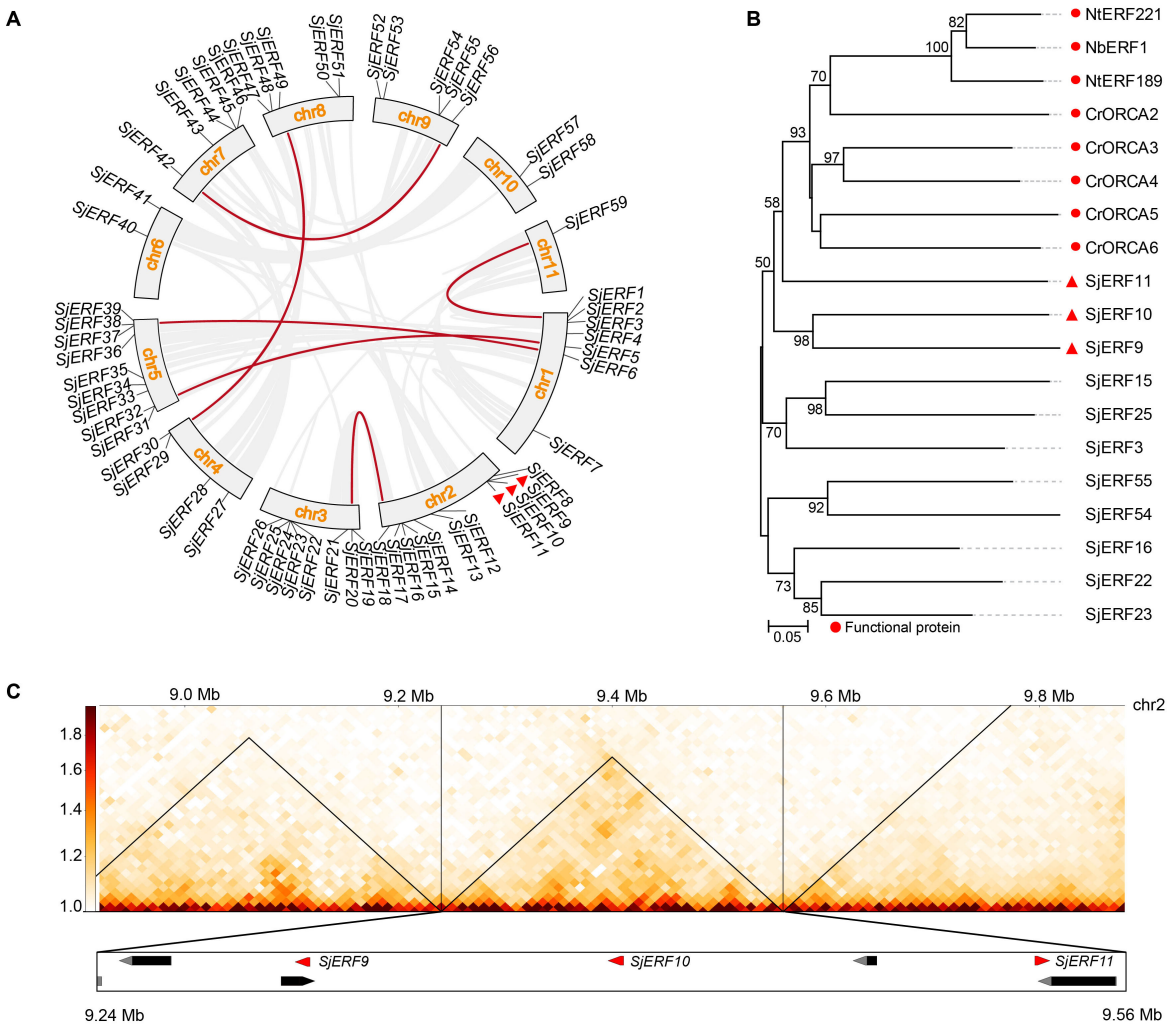
The chromosome distribution and synteny analysis of *SjERF*s. **(A)** Chromosomal locations and their synteny of *SjERF*s. The connecting lines indicate duplicated gene pairs in 59 *SjERF*s. **(B)** The phylogenetic tree of *SjERF*s and functional ERF cluster. **(C)** The topologically associating domains (TADs) region of three *SjERFs*.

Chromosome localization analysis found that 59 *SjERF*s were disproportionately distributed on eleven *S. japonica* chromosomes (**Figure 4A**). Seven *SjERF*s were distributed on Chr1 and Chr3, eleven *SjERF*s on Chr2, four *SjERF*s distributed on Chr4, nine *SjERF*s distributed on Chr5, two *SjERF*s distributed on Chr6 and Chr10, five *SjERF*s distributed on Chr7, Chr8 and Chr9, and only one *SjERF* distributed on Chr11. Interestingly, three *SjERF* genes containing *SjERF9*, *SjERF10*, and *SjERF11* were distributed on *S. japonica* chromosome 2 (9.30 - 9.54 Mb) and formed an *ERF* gene cluster. Similar results have also been found in *C. roseus* and *N. tabacum*, such as the *ORCA* gene cluster and *NICOTINE2* (*NIC2*) *ERF* cluster (Yuan, 2020; Shoji et al., 2010; Shoji and Yuan, 2021). The phylogenetic tree showed that *SjERF9*, *SjERF10*, and *SjERF11* and functional ERF cluster were converging into one branch, and belonging to the IX subfamily (**Figure 4B**). Additionally, three *SjERFs* and four other genes were located in the same topologically associating domains (TADs) region (**Figure 4C**). Overall, the *SjERF* gene cluster found in *S. japonica* genome may play an important role in the biosynthesis of secondary metabolism.

Through collinear analysis, the potential relationship and gene duplication type between *SjERF* genes in the *S. japonica* genome were explored (**Figure 4A**). A total of seven *SjERF* genes were found in three segmental duplication events, such as Chr1(*SjERF1*)/Chr11(*SjERF59*), Chr1(*SjERF5*)/Chr5(*SjERF31*), Chr1(*SjERF6*)/Chr5(*SjERF38*), Chr7(*SjERF42*)/Chr9(*SjERF56*), Chr4(*SjERF29*)/Chr8(*SjERF49*). To further infer the evolutionary mechanism of the *ERF* family in *S. japonica*, we constructed a synteny diagram of *S. japonica* with *C. roseus*, *N. tabacum*, *A. thaliana*, and *O. sativa* (**Supplementary Figure S5**). Between *S. japonica* and *A. thaliana*, *O. sativa*, *C. roseus*, and *N. tabacum*, 56, 46, 50, and 32 syntenic *SjERF* gene pairs were identified, respectively (**Supplementary Table S7**). Some *SjERF* genes had multiple orthologous gene pairs (one *SjERF* associated with multiple *AtERFs*). For instance, three synteny events occurred in three *SjERF*s, such as *SjERF9*, *SjERF30*, and *SjERF41.* Interestingly, the *SjERF1*, *SjERF8*, *SjERF9*, *SjERF11*, *SjERF26*, *SjERF29*, *SjERF30*, *SjERF38*, *SjERF44*, *SjERF46*, *SjERF49*, and *SjERF51* genes exhibited a conserved homologous relationship across all four species, suggesting that they might play a significant role in plant function.

### 3.6 *SjERFs* cluster specifically bind to the GCC-boxes in the promoters of CEP-associated genes *in vitro*

**Figure 5:**
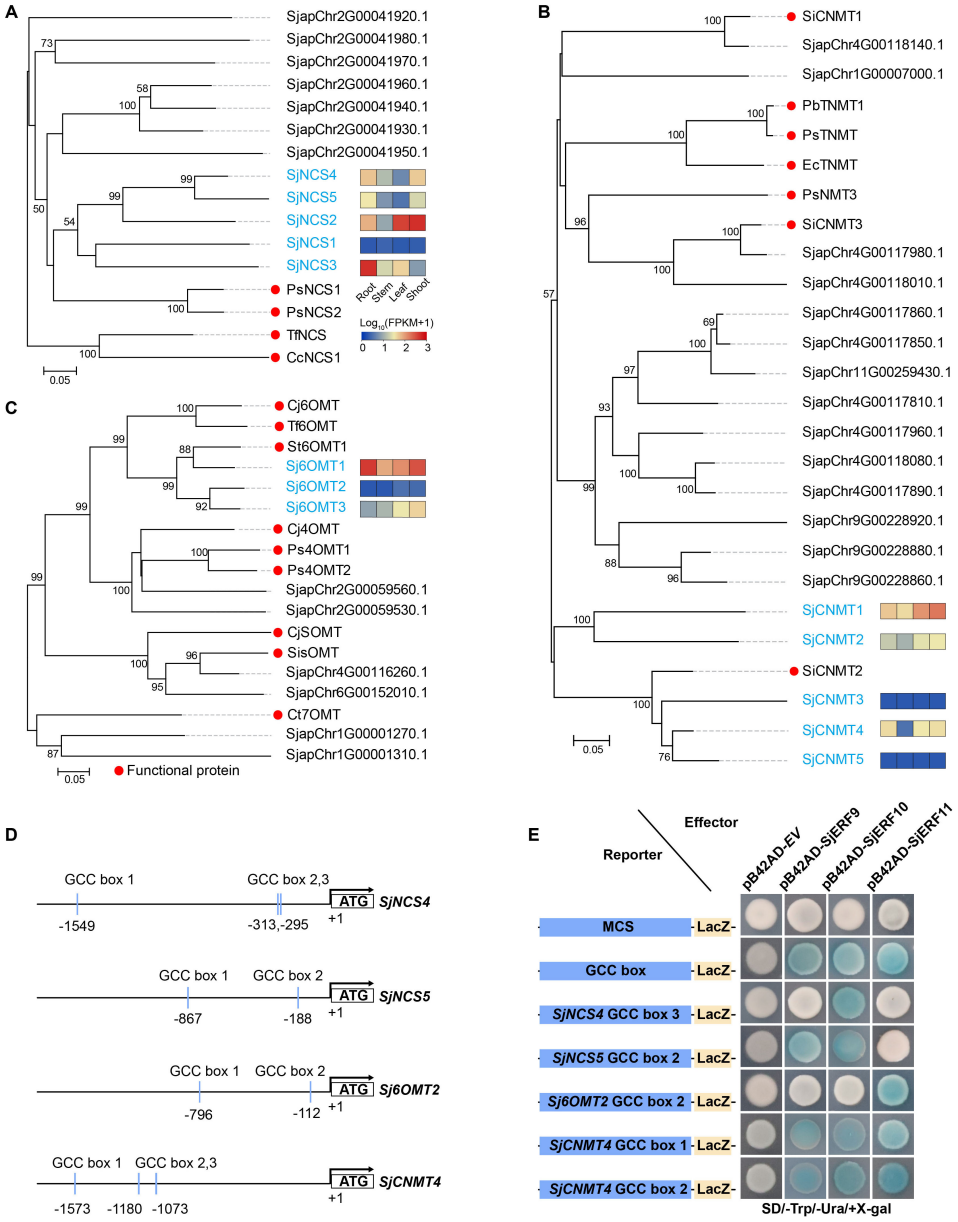
Members of the *SjERFs* cluster specifically bind to the GCC boxes in the promoters of CEP-associated genes *in vitro*. **(A–C)** Phylogenetic tree of CEP biosynthetic genes using MEGA11 with 1000 bootstrap replicates by Neighbor-joining (NJ) method. **(D)** Schematic diagrams of the *SjNCS4*, *SjNCS5*, *Sj6OMT2*, and *SjCNMT4* promoters. The positions of potential GCC boxes are shown as blue Rectangles. **(E)** Yeast one-hybrid (Y1H) assay indicates that the *SjERFs* cluster binds to the GCC box in the promoters of CEP-associated genes, including *SjNCS4*, *SjNCS5*, *Sj6OMT2* and *SjCNMT4*. Yeast cells transformed with different combinations of constructs were grown on SD/−Ura/−Trp/+X-gal medium. Photographs were taken after 3 d of incubation at 30°C. Y1H assays were repeated three times.

To predict the *SjERFs* involved in the CEP biosynthesis pathway, *NCS*, *6-OMT*, and *CNMT* genes were identified in the *S. japonica* genome using the BLASTP approach with *p*-value < 1e^-10^ (**Supplementary Table S2**). Subsequently, the well-supported subfamily containing the functional protein sequence was defined as candidate functional genes in the CEP biosynthesis pathway using a phylogenetic tree. Finally, five *NCS*, three *6-OMT*, and five *CNMT* genes were identified as candidate functional genes in *S. japonica* genome (**Figure 5**). Meanwhile, the majority of CEP-biosynthetic genes have high transcriptional levels in one or more tissues of *S. japonica*, except for *SjNCS1*, *SjCNMT3* and *SjCNMT5* (**Figure 5**; **Supplementary Table S8**). For instance, *Sj6OMT1*, *SjNCS3-5*, and *SjCNMT4* have the highest expression level in *S. japonica* root (FPKM >30), while *Sj6OMT3*, *SjNCS2*, and *SjCNMT1,2* exhibited preferential expression patterns in *S. japonica* shoots.

**Figure 6:**
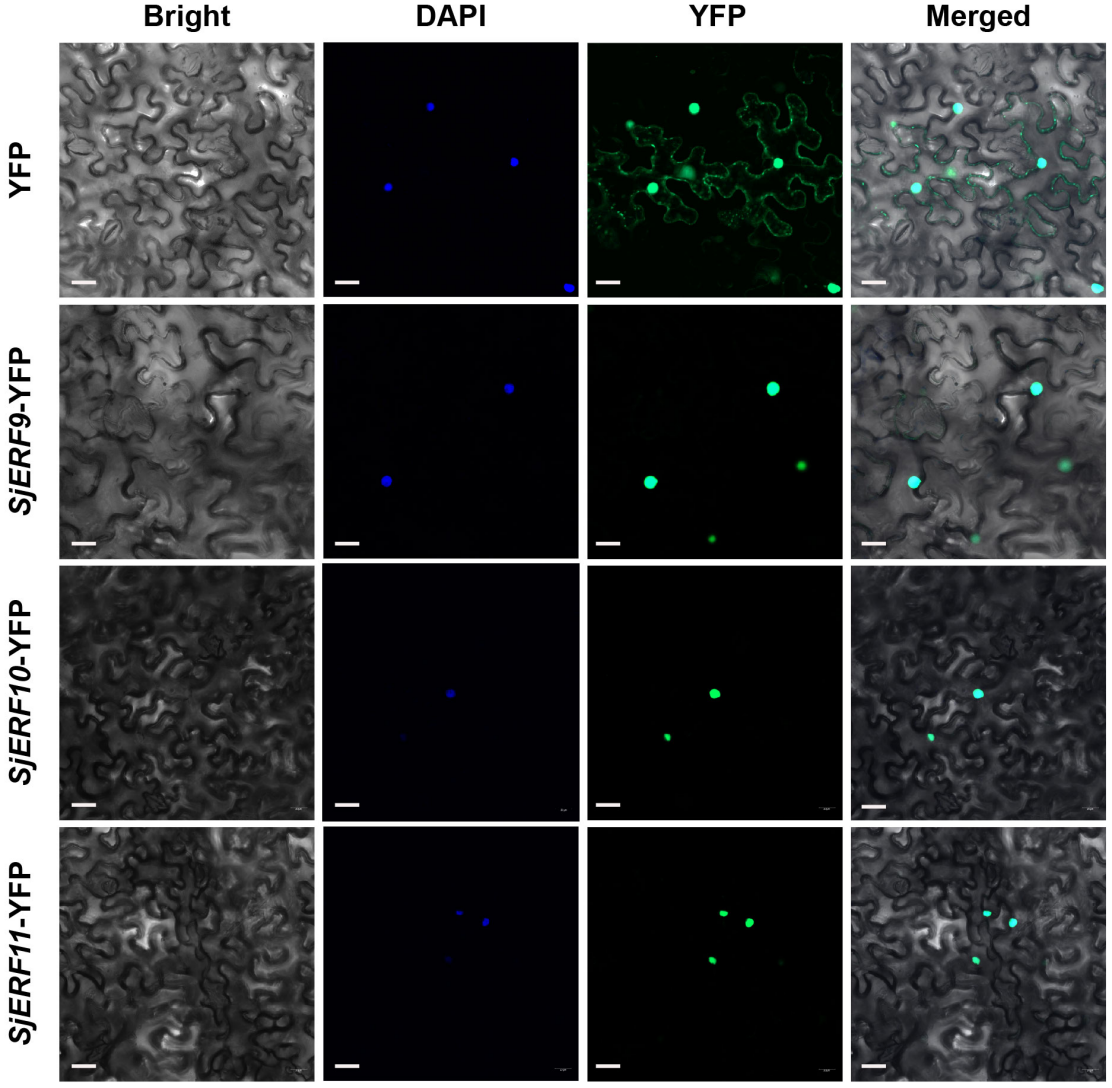
SjERFs protein fused to a yellow fluorescent protein (YFP) transiently expressed in *N. benthamiana*. Scale bar: 20 μm.

An increasing amount of data suggests that the *ERF* gene clusters play a crucial role in secondary metabolism (Paul et al., 2020; Shoji and Yuan, 2021). The *cis*-acting elements of CEP biosynthetic gene promoters were analyzed. Seven of the thirteen promoters (*SjNCS1*, *SjNCS3*, *SjNCS4*, *SjNCS5*, *Sj6OMT1*, *Sj6OMT2*, and *SjCNMT4*) contained either a predicted GCC motif or a GCC-like element (**Figure 5D**). Among them, three GCC-boxes were found in the promoter region of *SjNCS4* and *SjCNMT4*, whereas two GCC-boxes were identified in the *SjNCS5* and *Sj6OMT2* promoter. To further identify the *SjERFs* gene cluster involved in CEP biosynthesis, Y1H assays were carried out in this study. As depicted in **Figure 5E**, binding of the AD-SjERF9/10/11 (GAL4 AD-prey protein) fusion protein, but not AD-EV (GAL4 AD empty vector) alone, to three tandem repeats of the GCC-box, strongly activated the expression of the *Lac*Z reporter gene in the Y1H system. Moreover, the SjERF9 transcription factor regulates the expression of *SjNCS5* by directly binding the GCC-box2 of the *SjNCS5* promoter. SjERF10 could directly bind to the *SjNCS5* promoter, while SjERF11 recognizes GCC-box2 of the *Sj6OMT2* promoter. Interestingly, the SjERFs gene cluster was observed to bind to the GCC-box1, 2 in the promoter of *SjCNMT4*, indicating their potential crucial role in CEP metabolism. Additionally, the SjERFs gene cluster-YFP fusion proteins were observed exclusively in the nuclei, which is consistent with their putative role as transcription factors in the nucleus (**Figure 6**). In conclusion, our findings suggest that the SjERFs gene cluster regulates CEP biosynthesis by directly binding to the GCC-boxes in the promoters of CEP-associated genes.

### 3.7 Tissue expression profiling of *SjERF* TFs

**Figure 7:**
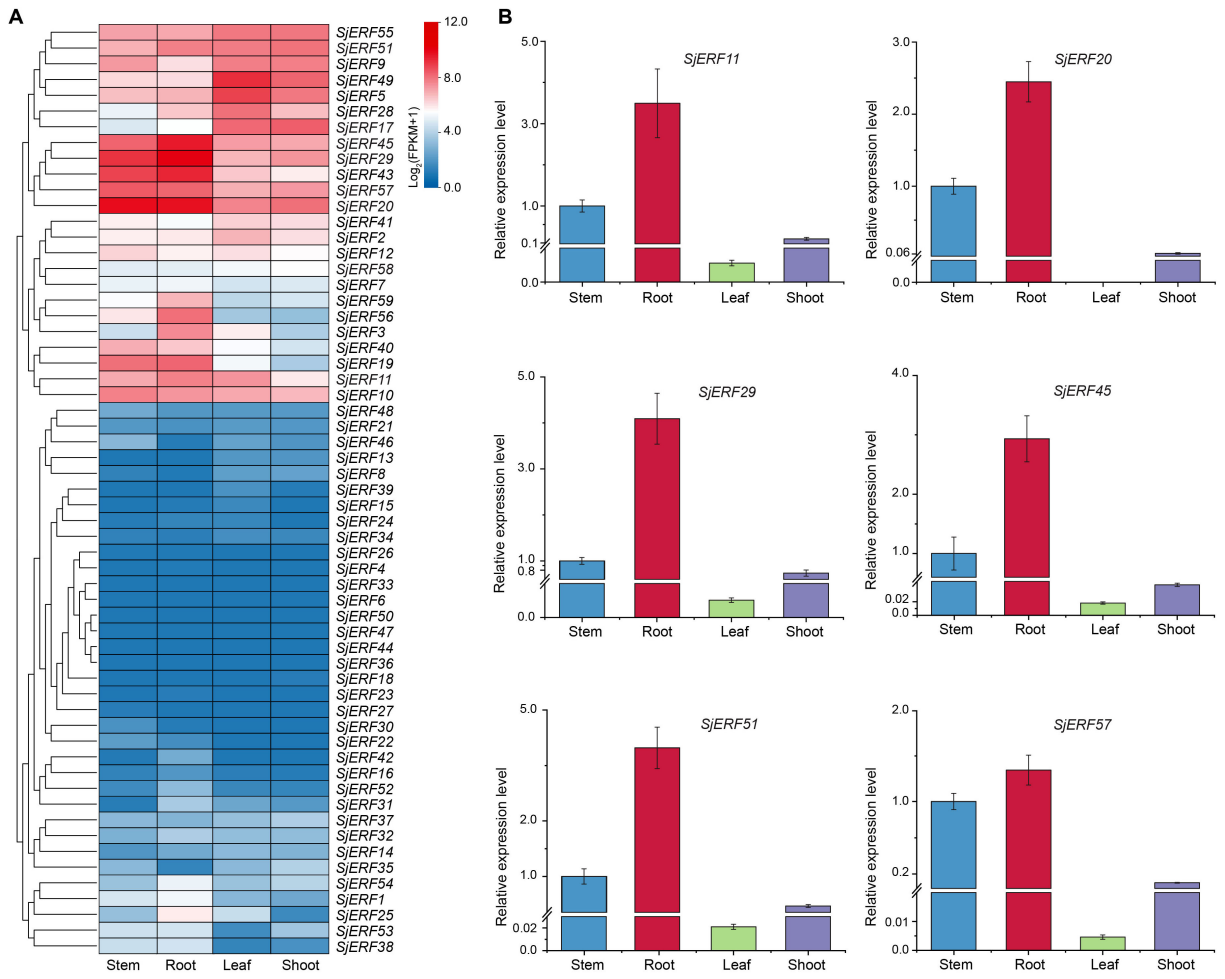
Expression patterns of 59 *SjERFs* in different tissues of *S. japonica.* **(A)** Hierarchical clustering of the expression level of *SjERFs* with RNA-Seq. **(B)** The expression profiles of six *SjERFs* in different tissues with the qRT-PCR method.

We analyzed the expression level of 59 *SjERFs* in roots, stems, leaves, and shoots of *S. japonica* from the available transcriptome data. Heat-map analysis showed that eleven *SjERF* genes were highly expressed in roots, stems, leaves, and shoots of *S. japonica* (FPKM > 50), including *SjERF5*, *9*, *17*, *20*, *29*, *43*, *45*, *49*, *51*, *55*, and *57* (**Figure 7A**). Among them, *SjERF20*, *29*, *43*, *45*, and *57* showed the highest expression level in *S. japonica* roots, and stems, respectively. However, thirteen genes had nearly no expression in the roots, stems, leaves, and shoots of *S. japonica* (FPKM < 1). Furthermore, some *SjERF* genes with tissue-specific or preferential expression patterns were observed in vegetative tissues of *S. japonica*. For example, *SjERF5* and *SjERF49* with the highest expressions were observed in *S. japonica* leaves. 13 *SjERFs* were observed with higher expression in root tissues of *S. japonica*. To validate the accuracy of RNA-seq, real-time qPCR was performed on six *SjERFs*, which exhibited significantly higher expression levels in the root of *S. japonica*. Overall, the results indicated that these *SjERFs* exhibited higher expression levels in the roots and lower expression in the leaves of *S. japonica* (**Figure 7B**). The qRT-PCR results of six *SjERFs* were consistent with the RNA-Seq data, indicating strong reliability of the RNA-Seq data.

### 3.8 Co-expression analyses of *SjERFs* involved in CEP biosynthesis

**Figure 8:**
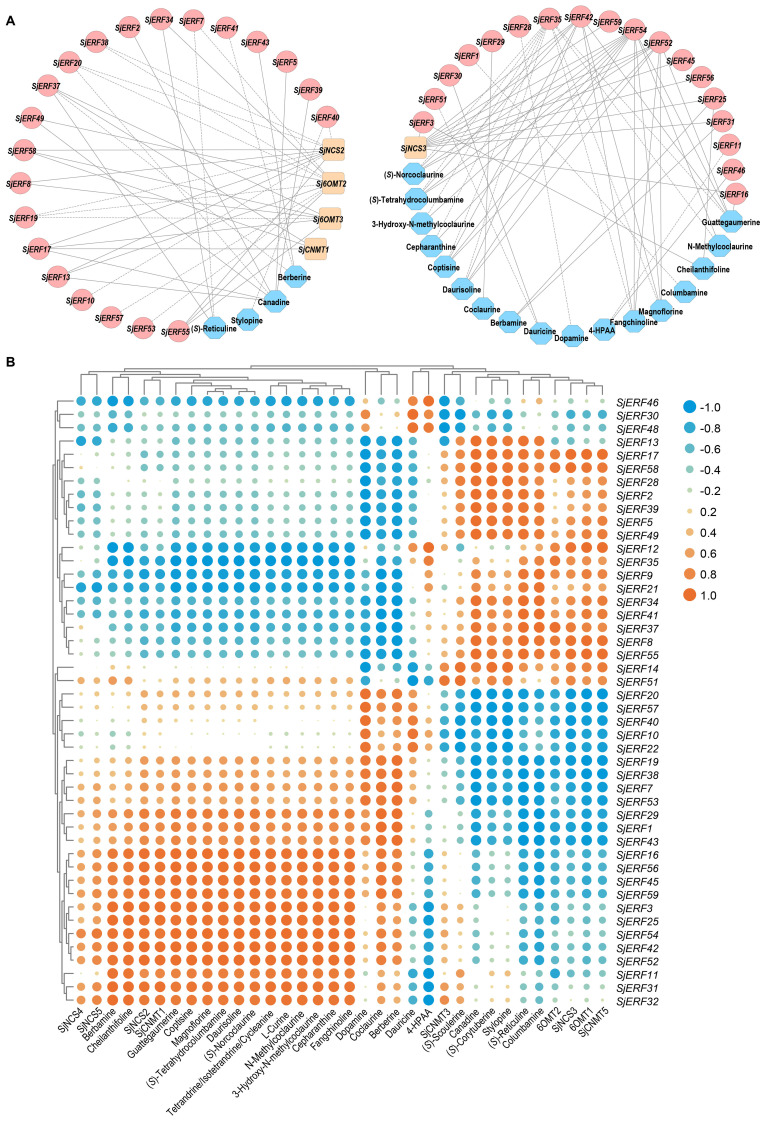
Analysis of correlation between *SjERFs*, CEP-biosynthetic genes, and BIAs metabolites. **(A)** Co-expression network of *SjERF* genes, CEP biosynthetic genes, and BIAs metabolites with |r| > 0.9 and *p*-value < 0.05. Orange squares: CEP biosynthetic genes, red circles: *SjERF* genes. Blue octagons: BIAs metabolites. **(B)** The cluster heatmap shows the expression correlations between five CEP biosynthetic genes, and two BIA precursors, alongside 23 BIA-type structures, and forty-six *SjERFs*.

Co-expression analysis of SjERFs, CEP biosynthetic genes, and BIAs metabolites was visualized using the Cytoscape tool. The co-expression network analysis revealed a strong correlation between the expression levels of 35 *SjERFs* and CEP biosynthetic genes in *S. japonica* (Pearson correlation coefficient r > 0.9 and *p*-value < 0.05). It is worth noting that *SjERF17* and *SjERF58* were strongly positively correlated with the three CEP biosynthetic genes, respectively (**Figure 8**; **Supplementary Table S9**). The expression profile of *SjERF10* was correlated strongly with *SjNCS2* and *SjCNMT2* genes. Additionally, *SjERF54* was highly positively correlated with ten BIAs, while *SjERF35* was highly negatively correlated with these metabolites, including (*S*)-Norcoclaurine, N-Methylcoclaurine, 3-Hydroxy-N-methylcoclaurine, Magnoflorine, Coptisine, (*S*)-Tetrahydrocolumbamine, Guattegaumerine, Daurisoline, Fangchinoline, and Cepharanthine. *SjERF42* and *SjERF52* were highly positively correlated with seven BIAs. In summary, these findings suggest that these *SjERFs* may be involved in the biosynthesis of CEP and its precursors.

## 4 Discussion

The *AP2/ERF* gene family is a plant-specific group of transcription factors, characterized by an AP2 domain for DNA binding (Licausi et al., 2013). Typically, members of this family usually contain one to two highly conserved AP2 domains. The AP2 subfamily members consist of members with two repeated AP2 domains, while the *ERF* subfamily contains members with a single AP2 domain (Sakuma et al., 2002). *ERF* transcription factors have significant effects in regulating the biosynthesis of the main pharmaceutical active components in medicinal plants (Gu et al., 2017; Wang M. et al., 2021). Extensive research on the *ERF* family has been conducted in various plants, including soybean, tomato, apple, corn, barley, and common wheat (Gu et al., 2017; Feng et al., 2020). However, genome-wide identification of ERF protein in BIA-producing plants remains limited.

In this study, 59 *SjERFs* have been identified in the *S. japonica* genome (**Table 1**; **Figure 1**), which is similar to the 60 *EcERF* genes in *E. californica* (Yamada et al., 2020), 59 in *Cannabis sativa* (Tian et al., 2020), and 65 in *Spirodela polyrhiza* (Tian et al., 2020). Each of these ERF genes is characterized by a single conserved AP2 domain. Notably, the number of *SjERF* gene members in the *S. japonica* genome was less than *Oryza sativa* (139 genes), *Zea mays* (136), *A. thaliana* (122), *Glycine max* (122), and *Triticum aestivum* (99) (Nakano et al., 2006; Feng et al., 2020). Collinear analysis was performed to explore the potential relationships between the *SjERF* genes in the *S. japonica* genome. The analysis revealed that a total of twelve *SjERF* genes were involved in six segmental duplication events (**Figure 4**). These segmental duplication events have likely contributed to the expansion of the ERF family in *S. japonica*.

Two commonly used classification systems were established in *A. thaliana* (Riechmann and Meyerowitz, 1998; Nakano et al., 2006). Riechmann et al. classified 144 *AP2/ERF* transcription factors into three classes (Riechmann and Meyerowitz, 1998); Sakuma et al. divided 145 *AP2/ERF* transcription factors into five classes and further divided the *DREB* subfamily into six subgroups (A1 to A6), and the *ERF* subfamily into six subgroups (B1 to B6) (Sakuma et al., 2002). In contrast, Nakano et al. classified *ERF* subfamily transcription factors into ten groups, which were named groups I to X, instead of the two major subfamilies (*DREB* and *ERF*) (Nakano et al., 2006). A phylogenetic tree of this work showed that 59 *SjERFs* were further categorized into ten subfamilies based on 121 *AtERFs* (**Figure 1**). This classification was in harmony with the evolutionary analyses of Nakano et al (Nakano et al., 2006). Generally, *ERFs* within the same group exhibit evolutionary conservation and share similar gene structures (Cao et al., 2020). Gene structure analysis revealed that 67.8% of *SjERFs*, including those from group II and III, contained only one exon, indicating a conserved gene structure for most *SjERFs* (**Figure 2C**). These findings were consistent with pineapple, where 66.22% of *AcERFs* displayed a similar gene structure (Huang et al., 2020). Furthermore, *cis*-element analysis of the promoter regions demonstrated that the majority of *SjERF* genes were involved in light-responsive processes (119), phytohormone responses (ABA, MeJA) (253), as well as abiotic and biotic stress responses (565) (**Figure 3**). Specifically, 164 abscisic acid responsiveness *cis*-acting regulatory elements (ABREs) were detected in the promoter regions of 57 *SjERFs*, excluding *SjERF57* and *SjERF59*. Additionally, MeJA-responsive elements were discovered in the promoter regions of 54 *SjERF* genes, including *SjERF* gene clusters. Previous studies demonstrated that JAs are key signaling molecules involved in alkaloid biosynthesis (Wang M. et al., 2021). Many *ERF* transcription factors respond to jasmonic acid and activate the expression of alkaloid-associated genes, such as *CrORCA* and *NbERF189* (van der Fits and Memelink, 2000; Shoji et al., 2010). Thus, these findings indicated that *SjERFs* can be regulated by various *cis*-acting elements in their promoters during growth and stress responses.

ERF TFs not only affect plant growth and development but also play a crucial role in secondary metabolisms, such as terpenoids, phenylpropanoids, and alkaloids (Zhou and Memelink, 2016; Shoji and Yuan, 2021; Godbole et al., 2022). The majority of ERFs shown to participate in secondary metabolites biosynthesis are members of group IX. Several group IX AP2/ERFs form physically linked gene clusters and have been characterized in a limited number of plant species, including *Nicotiana tabacum* (Kajikawa et al., 2017), potato (Cárdenas et al., 2016), and *C. roseus* (Paul et al., 2017). For instance, *AaORA* positively regulates artemisinin biosynthesis in *Artemisia annua* and activates the expression of *AaADS*, *AaCYP71AV1*, and *AaDBR2* (Lu et al., 2013). In *C. roseus*, the ORCA cluster, consisting of ORCA3, ORCA4, and ORCA5, is a crucial regulator in alkaloid biosynthesis (van der Fits and Memelink, 2000; Singh et al., 2020). It is also proved that ERF189, ERF221, and the NIC2-locus clustered ERFs in *N. benthamiana* activate the nicotine biosynthetic pathway by affecting several nicotine biosynthetic genes (Shoji et al., 2010). To date, only the genome-wide identification and systematic analysis of ERF transcription factors in *E. californica*, which produces BIA, have been completed. It has been found that four Group IX ERFs can activate the expression of key enzyme genes involved in BIA biosynthesis (Yamada et al., 2020). In *Coptis chinensis*, *cis*-acting elements of BIA biosynthetic gene promoters were conducted and showed the involvement of GCC-box and ERF transcription factors in the regulation of berberine biosynthesis (Yamada et al., 2016). Nonetheless, the role of the ERF subfamily in CEP biosynthesis remains unexplored. In the present study, the co-expression network between *SjERF*s, CEP-associated genes, and BIAs metabolites showed that *SjERF17* and *SjERF58* have a strong correlation with the expression levels of CEP biosynthetic enzyme genes. *SjERF42* and *SjERF52* show positive correlations with the content of seven BIA metabolites; These results suggested that they might be involved in regulating the biosynthesis of CEP and its precursors (**Figure 8**). Notably, an *ERF* cluster (*SjERF9/10/11*) has also been identified in the *S. japonica* genome and is localized to the nucleus, respectively. Yeast one-hybrid assays proved that three *SjERFs* could directly bind to several CEP biosynthetic genes, including *SjCNMT4* (**Figure 5**). In summary, the findings of this study indicate that *SjERF* cluster may act as a direct regulator of CEP metabolism by regulating the expression of CEP-associated genes. This study provides a foundation for analyzing the underlying molecular mechanism of CEP biosynthesis and further investigating the functional genomics of candidate *SjERF* genes.

## 5 Conclusions

This is the first study that 59 *SjERF*s were identified and categorized into ten subfamilies in *S. japonica* genome. Through a series of bioinformatics analyses of 59 *SjERF*s, it was found that the gene structure of *SjERF32*, and *SjERF54* in same group was highly similar. Through collinear analysis, we identified twelve *SjERF* genes from the *ERF* genome data of *S. japonica* that were involved in six segmental duplication events. One gene cluster containing three *SjERF* genes was found on chromosome 2, which is close to the evolution of functional *ORCA* genes in *C. roseus*. Furthermore, the *SjERFs* cluster was observed to bind to the CEP-associated gene promoters, suggesting that the *SjERFs* cluster may act as a direct regulator of CEP metabolism. The tissue expression profile revealed that most *SjERF* genes were highly expressed in *S. japonica* root. Furthermore, we constructed a co-expression network between *SjERF*s, CEP biosynthetic genes, and BIAs metabolites, and several *SjERFs* were highly positively correlated with the contents of diverse BIAs of *S. japonica*. These results provide a basis for further characterizing the biological function of the *SjERF* gene and analyzing its molecular mechanism of regulating CEP biosynthesis.

## Data Availability

The raw data of Genome and RNA-seq data sets for the transcriptome analysis are available in NCBI, under BioProject PRJNA888087.
