# Supplementary material for: Genome-wide analysis of the ERF Family in Stephania japonica provides insights into the regulatory role in Cepharanthine biosynthesis
Source: Front Plant Sci. 2024 Sep 4;15:1433015. doi: 10.3389/fpls.2024.1433015 (PMC11408324; doi:10.3389/fpls.2024.1433015)
Supplement: Supplementary file 1 [file DataSheet1.docx]

Supplementary Material


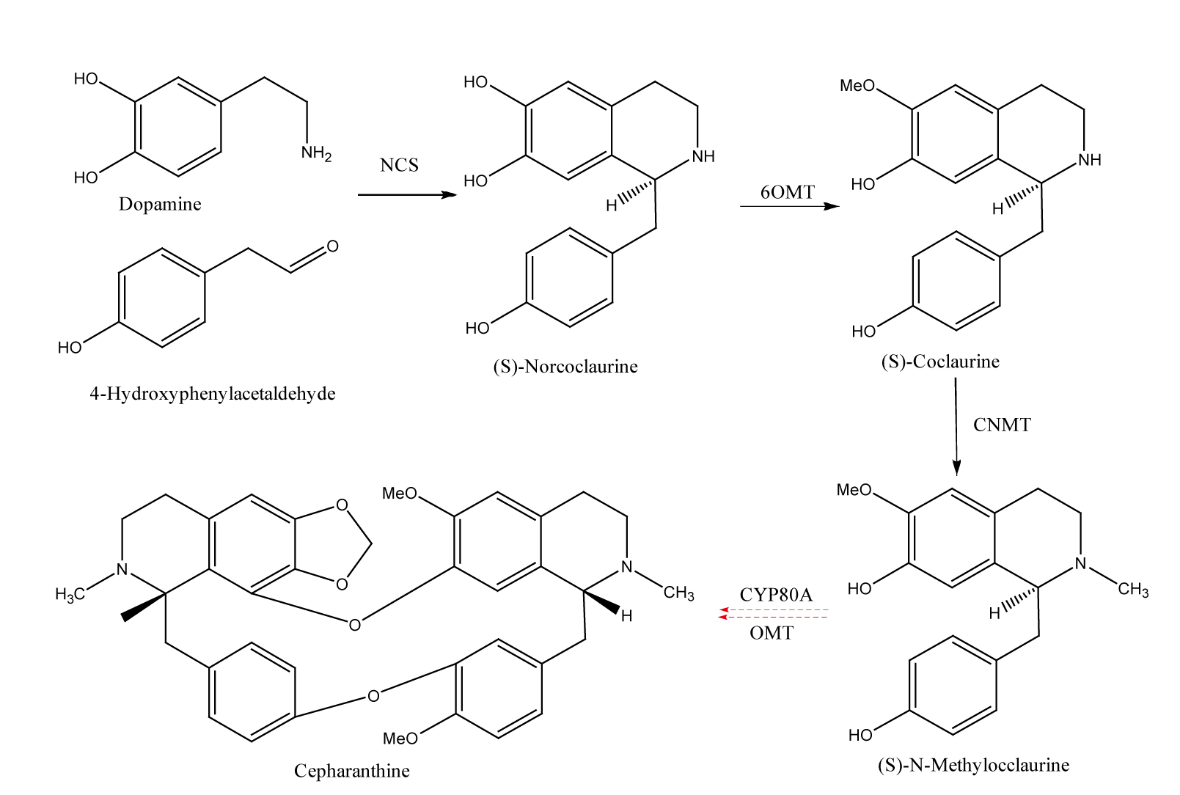


Fig. S1. Cepharanthine biosynthesis pathway.


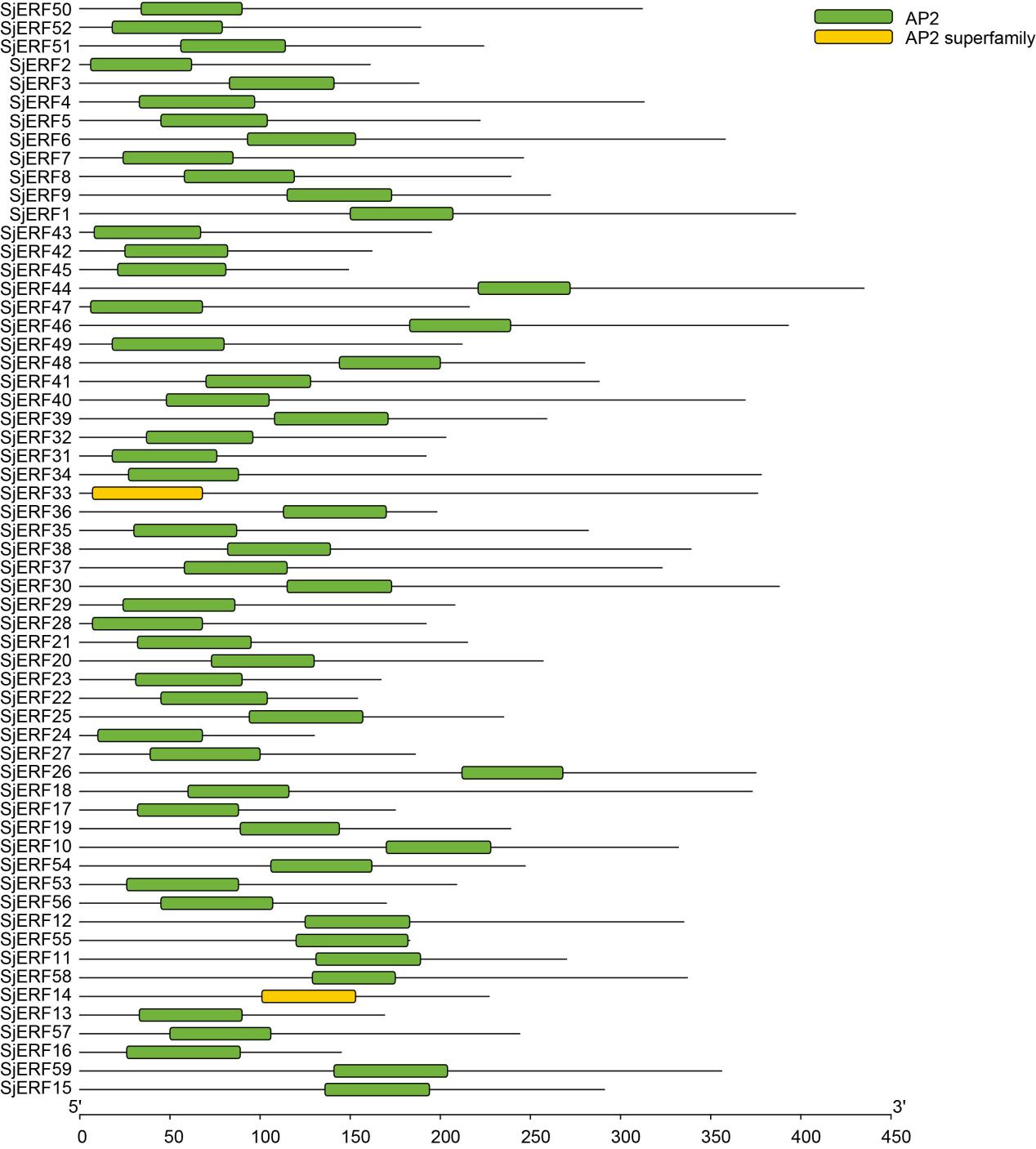


Fig. S2. Conserved domain of SjERF proteins.


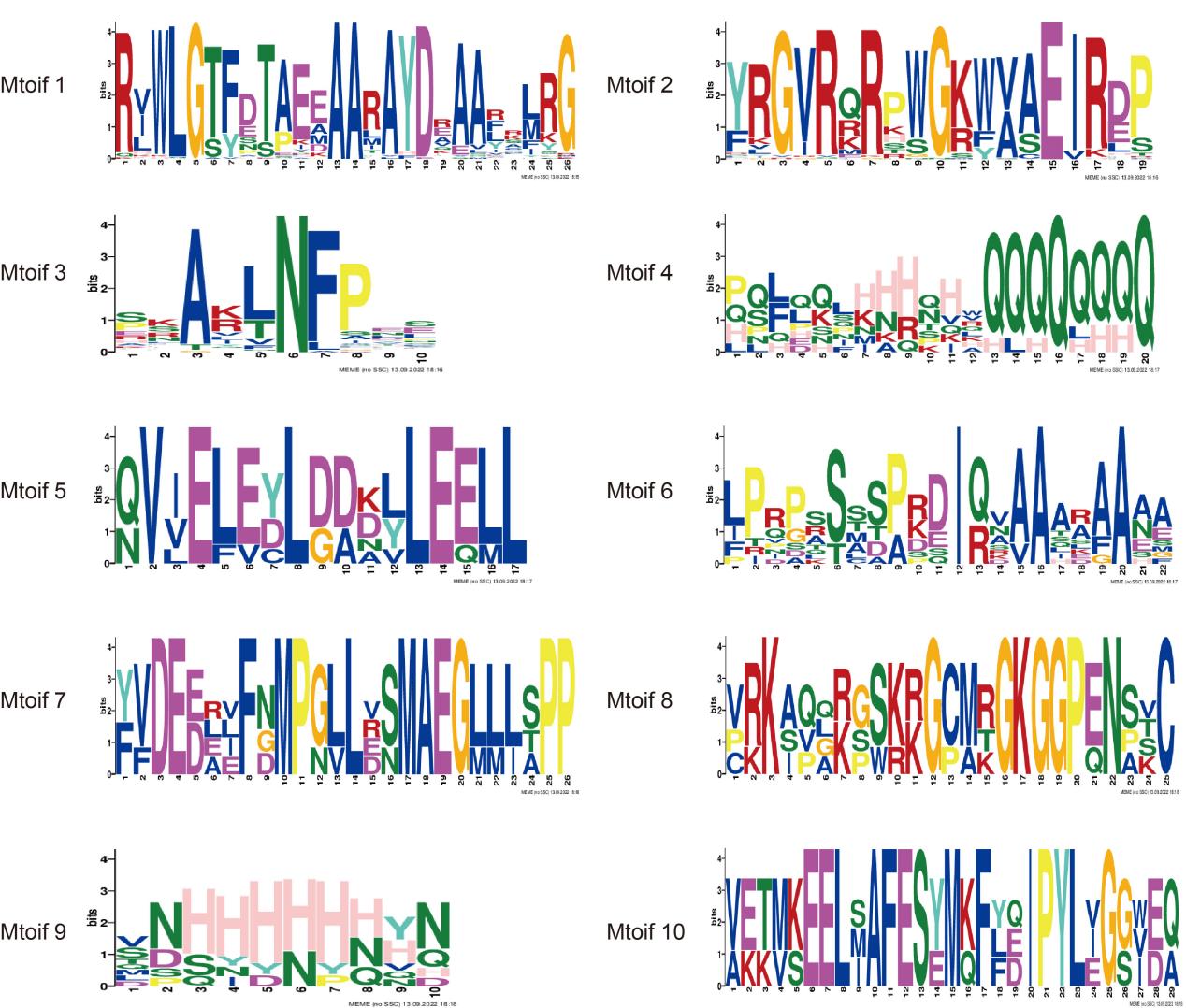


Fig. S3. The seqlogos of motif of the SjERFs proteins.


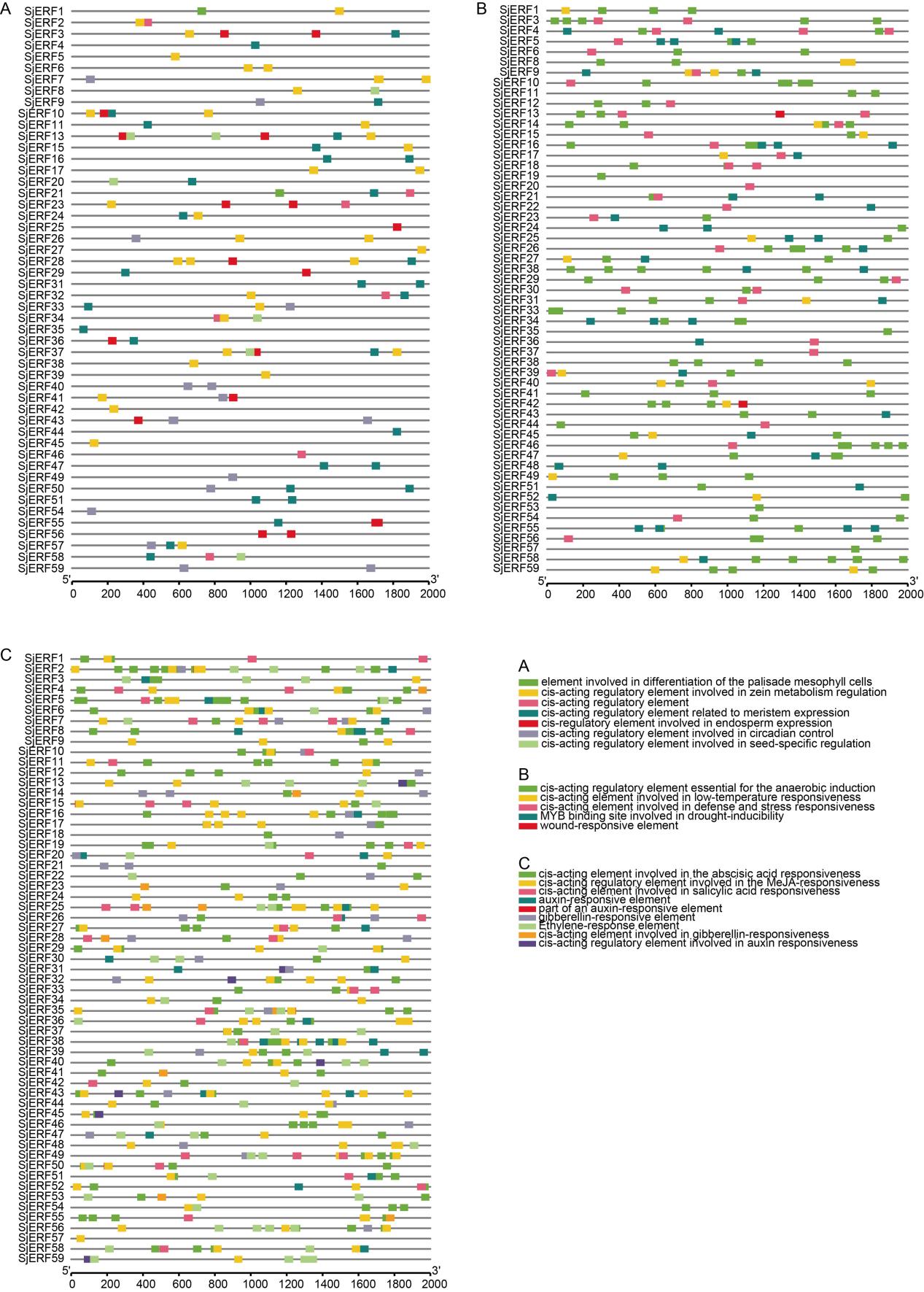


Fig. S4. Schematic representations of *cis*-regulatory elements present in SjERFs as investigated via the PlantCARE tool. (A) Plant growth and development. (B) Abiotic and biotic strees. (C) Phytobormone responsive.


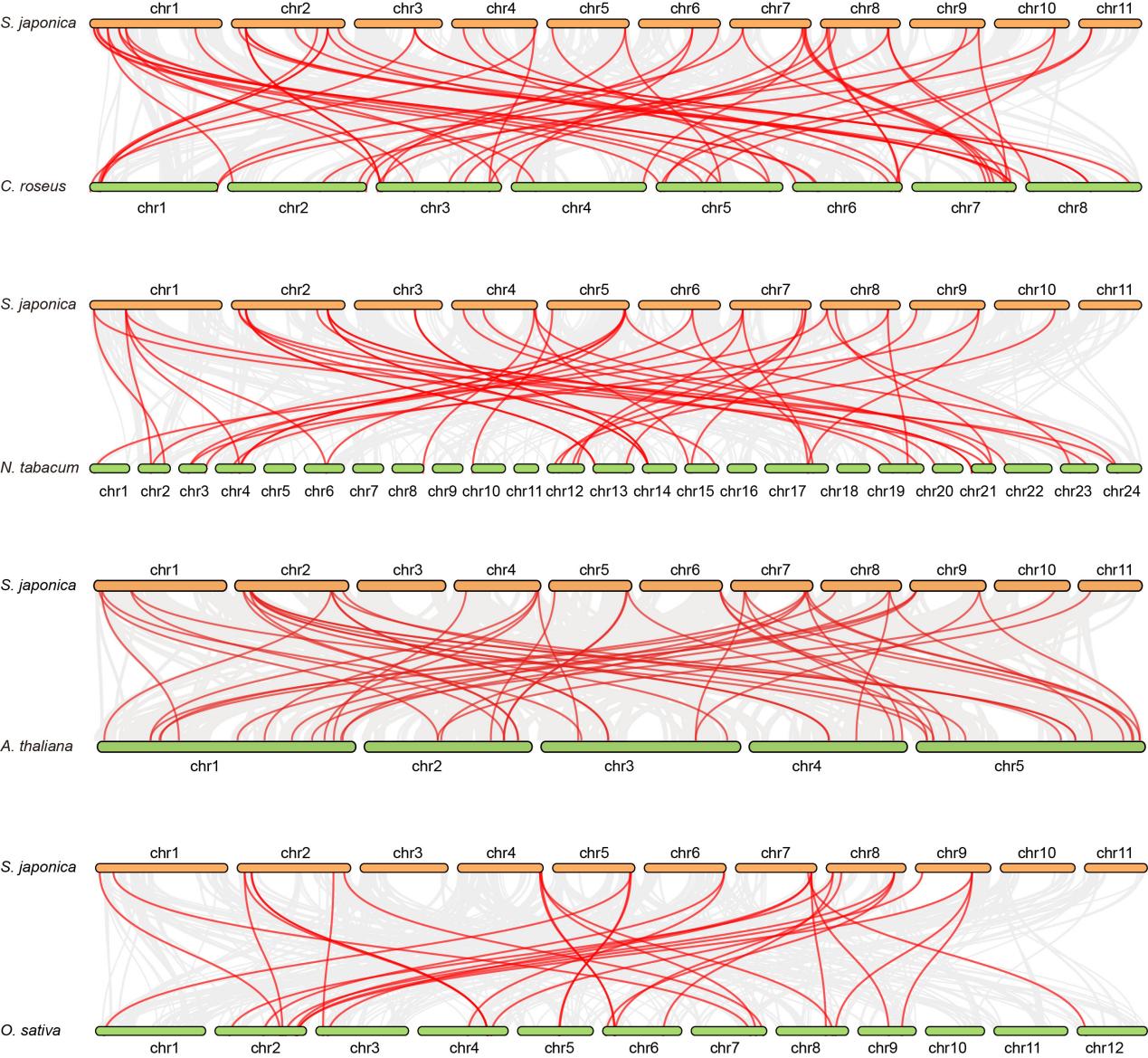


Fig. S5.The Synteny analysis of SjERF genes between *S. japonica* with

*C. roseus, N. tabacum**, A. thaliana and O. sativa.*
